# Fabrication and Biological Assessment of Antidiabetic α-Mangostin Loaded Nanosponges: In Vitro, In Vivo, and In Silico Studies

**DOI:** 10.3390/molecules26216633

**Published:** 2021-11-01

**Authors:** Faisal Usman, Hamid Saeed Shah, Sumera Zaib, Sirikhwan Manee, Jahanzeb Mudassir, Ajmal Khan, Gaber El-Saber Batiha, Khamael M. Abualnaja, Dalal Alhashmialameer, Imtiaz Khan

**Affiliations:** 1 Department of Pharmaceutics, Faculty of Pharmacy, Bahauddin Zakariya University, Multan 66000, Pakistan; faisal.usman@bzu.edu.pk (F.U.); jahanzebmudassir@bzu.edu.pk (J.M.); 2 Institute of Pharmaceutical Sciences, University of Veterinary and Animal Sciences, Lahore 54000, Pakistan; 3 Department of Biochemistry, Faculty of Life Sciences, University of Central Punjab, Lahore 54590, Pakistan; 4 Faculty of Traditional Thai Medicine, Prince of Songkla University, Hat-Yai, Songkhla 90110, Thailand; sirikhwan.m@gmail.com; 5 Natural and Medical Sciences Research Center, University of Nizwa, Nizwa 616, Oman; ajmalkhan@unizwa.edu.om; 6 Department of Pharmacology and Therapeutics, Faculty of Veterinary Medicine, Damanhour University, Damanhour 22511, Albeheira, Egypt; gaberbatiha@gmail.com; 7 Department of Chemistry, College of Science, Taif University, Taif 21944, Saudi Arabia; k.ala@tu.edu.sa (K.M.A.); dsamer@tu.edu.sa (D.A.); 8 Department of Chemistry and Manchester Institute of Biotechnology, The University of Manchester, 131 Princess Street, Manchester M1 7DN, UK

**Keywords:** diabetes, drug delivery, α-glucosidase, in vivo studies, α-mangostin, molecular docking, nanosponges, quasi-emulsion method

## Abstract

Type 2 diabetes mellitus has been a major health issue with increasing morbidity and mortality due to macrovascular and microvascular complications. The urgent need for improved methods to control hyperglycemic complications reiterates the development of innovative preventive and therapeutic treatment strategies. In this perspective, xanthone compounds in the pericarp of the mangosteen fruit, especially α-mangostin (MGN), have been recognized to restore damaged pancreatic β-cells for optimal insulin release. Therefore, taking advantage of the robust use of nanotechnology for targeted drug delivery, we herein report the preparation of MGN loaded nanosponges for anti-diabetic therapeutic applications. The nanosponges were prepared by quasi-emulsion solvent evaporation method. Physico-chemical characterization of formulated nanosponges with satisfactory outcomes was performed with Fourier transform infra-red (FTIR) spectroscopy, differential scanning calorimetry (DSC), and scanning electron microscopy (SEM). Zeta potential, hydrodynamic diameter, entrapment efficiency, drug release properties, and stability studies at stress conditions were also tested. Molecular docking analysis revealed significant interactions of α-glucosidase and MGN in a protein-ligand complex. The maximum inhibition by nanosponges against α-glucosidase was observed to be 0.9352 ± 0.0856 µM, 3.11-fold higher than acarbose. In vivo studies were conducted on diabetic rats and plasma glucose levels were estimated by HPLC. Collectively, our findings suggest that MGN-loaded nanosponges may be beneficial in the treatment of diabetes since they prolong the antidiabetic response in plasma and improve patient compliance by slowly releasing MGN and requiring less frequent doses, respectively.

## 1. Introduction

**Figure 1:**
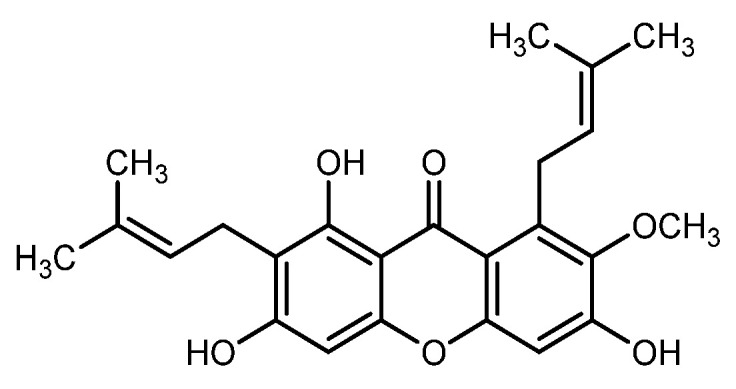
The chemical structure of mangostin.

*Garcinia mangostana* Linn., more commonly known as mangosteen (MG), belongs to the family Guttiferae [1,2]. The genus *Garcinia* is native to South-East Asian countries comprising more than 300 distinct species, with each family reported for distinct bioactive compounds such as xanthones, flavonoids, triterpenoids, and benzophenones [3,4]. The mangosteen pericarp possesses a therapeutically active compound named mangostin (MGN) (Figure 1). The two abundant forms (α and γ) are present in the pericarp and xanthone constitutes the core structure of MGN [5,6]. Besides showing antihypertensive and anti-inflammatory potential, xanthone molecule has proved more effective than vitamin C and E in combating reactive oxygen species that are involved in cell damage [7,8,9]. The xanthone compounds, especially MGN, have been recognized to meliorate damaged pancreatic β-cells for optimal insulin release [10,11,12,13,14,15,16].

Type 2 diabetes mellitus (T2DM) with its macrovascular and microvascular complications represent a major wide-reaching health warning with growing morbidity and mortality rate [17]. According to the International Diabetes Federation (IDF), 463 million adults have developed T2DM in 2019 worldwide [18], and this estimate is projected to upsurge to 700 million by 2045 [19]. The largest escalation will come from the countries undergoing monetary evolutions from low-income to middle-income tiers [20]. Over the past few years, natural sources were of high therapeutic interest to avert the impact of free radicals on cells which may be useful in treating various metabolic diseases [21,22,23,24,25].

In recent years, a liaison between phytochemistry and therapeutics has escalated the health-promoting benefits of various plants and their products [26]. The major obstacle of directly utilizing herbal extracts for treating various diseases is their failure to precisely traverse the lipid bilayer that may lead to the impecunious bioavailability of therapeutic agents [27,28]. However, delivering a drug with nanotechnology may improve the bioavailability and therapeutic efficiency of phytochemicals [29,30,31]. Nanosponges are highly porous nanoparticles composed of hyper-cross-linked polymers and can entrap active molecules. The porous architecture of the nanosponges provides superior drug absorption/complexation properties [32,33]. These tiny structures have the capability of encapsulation of both hydrophilic and hydrophobic drugs and can enhance the solubility of poor water-soluble molecules [34,35,36]. Their particle size also offers a favorable environment for sustained drug release action for temporal and targeted purposes [37,38].

To the best of our knowledge, no MGN has previously been embedded within a nanosponge system offering a new delivery method for antidiabetic MGN. The goal is to maximize the therapeutic effects of MGN by releasing it in a sustained manner via nanosponges. Because the MGN is not instantly available, the possibility of harmful consequences by over-sensitizing insulin-producing cells could be minimized. The prepared nanosponges were characterized for their hydrodynamic diameter, surface analysis, encapsulation efficiency, and release potential. In vitro enzymatic inhibition, in vivo anti-diabetic effect, and in silico assessment were also carried out to provide an in-depth picture of the targeted therapy.

## 2. Results and Discussion

### 2.1. Physical Characterization

#### 2.1.1. Fourier-Transform Infra-Red (FTIR) Spectroscopic Analysis

**Figure 2:**
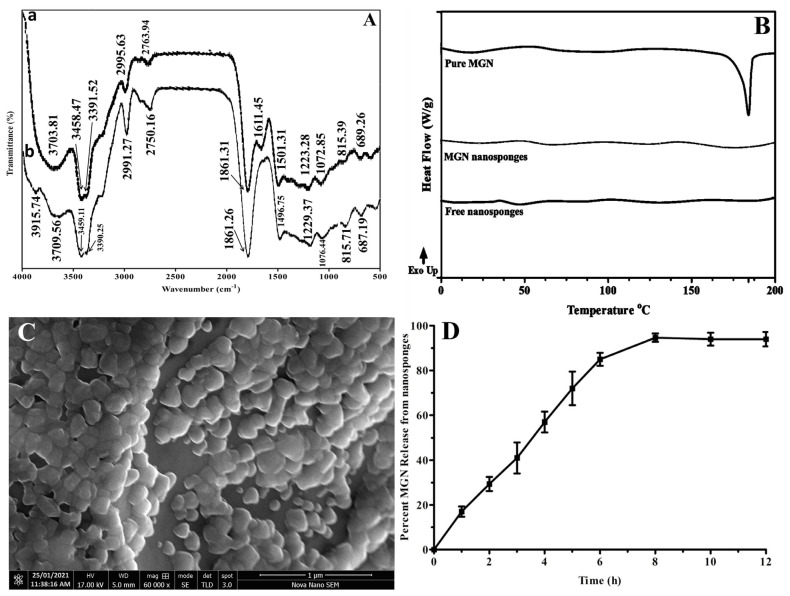
Physico-chemical characterization of prepared MGN nanosponges concerning FTIR (**A**) where spectrum (a) represents pure MGN while (b) shows MGN nanosponges, DSC (**B**), scanning electron microscopy (**C**), and MGN release from nanosponges (**D**).

FTIR spectra of pure MGN and its nanosponges are shown in Figure 2A. In MGN nanosponges (b), a low wide peak at 3915.74 cm^−1^ has been observed, attributable to the presence of trace amounts of water molecules, that may have played a role in hydrogen bonding. Due to O–H stretching, a typically weak and broad peak appeared in the spectra of both pure MGN (3703.81 cm^−1^) and MGN loaded nanosponges (3709.56 cm^−1^). Strong and steep peaks were seen as a result of intermolecular O–H stretching at wavenumbers (3458.47 and 3391.52 cm^−1^) in pure MGN spectrum and wavenumbers (3459.11 and 3390.25 cm^−1^) in the MGN nanosponges spectrum. The =C–H group stretching vibrations generated small peaks in the MGN spectrum at 2995.63 cm^−1^ whereas the identical peaks were also observed in MGN nanosponges with a minor shift towards lower wavelength (2991.27 cm^−1^). Additionally, a redshift in the functional group area of the MGN nanosponges (2750.16 cm^−1^) was observed due to O–H stretching, which was similarly seen in pure MGN (2763.94 cm^−1^). Due to C=O stretching, a weak and sharp peak emerged in the spectra of pure MGN (1611.45 cm^−1^), while MGN loaded nanosponges were devoid of this peak due to the possible contact with excipients (EC and PVA). In addition, the presence of C=C aromatic ring (1501.31 and 1496.75 cm^−1^), C–O–C bond (1223.28 and 1229.37 cm^−1^), and C–OH bending vibrations (1072.85 and 1076.44 cm^−1^) was verified in MGN and MGN loaded nanosponges, respectively. The FTIR data for MGN were consistent with earlier reported results [39,40].

#### 2.1.2. Differential Scanning Calorimetric (DSC) Analysis

DSC gives important information on the drug’s thermal behavior, structural alterations, crystallinity, and interaction with excipients [41]. Thermal imaging of pure MGN and MGN nanosponges was evident for compatibility among drugs and formulation excipients. As demonstrated in Figure 2B, the MGN melting point (T_m_) peak was spotted at 183 °C. The characteristic melting point (T_m_) peak in the thermogram of MGN nanosponges was disappeared representing the conversion from crystalline to amorphous form inside the nanosponges. The amorphous form of a drug substance improves its solubilization due to increased internal energy and reduction in thermodynamic stability, without affecting its medicinal properties and conformance with its excipients [42,43].

#### 2.1.3. Scanning Electron Microscopic (SEM) Analysis

The physical properties of nanosponges are dependent on the type of excipients used in the formulation [44]. The preparation of nanosponges utilizing the quasi-emulsion solvent evaporation technique mostly gives nanosponges with spherical shapes [45]. The MGN nanosponges portrayed in Figure 2C were characterized by a porous surface that was related to the degree of DCM diffusion from the surface as evident from previous reports [46,47,48]. It is conspicuous that the lower concentrations of EC and PVA led to better diffusion of the internal phase (dichloromethane) into the exterior phase (aqueous phase), which resulted in a reduction in the time required for the formation of porous structure [49,50,51,52,53,54].

#### 2.1.4. Nanosponges Size Analysis

The hydrodynamic diameter, zeta potential, and polydispersity index (PDI) of MGN nanosponges were estimated as 113 ± 8 nm, −35.06 ± 4.91 mV, and 0.3890 ± 0.0943, respectively (Table 1). It is evident that increasing the polymer and surfactant (EC and PVA) percentage resulted in a substantial increase in particle size owing to foaming and aggregation [37,44,47]. The zeta potential is influenced by the Brownian motion of suspended particles, and a high scale of zeta potential is associated with better stability of the dispersion [49]. Moreover, viscosity of the system can be improved as the amount of EC in the system is increased, making it more difficult to produce fine dispersion [38,55].

In this study, the measurements of zeta potential displayed a reasonable negative charge value −35.06 ± 4.91 mV that revealed an electrostatic stabilization on the surface of nanosponges [50]. A PDI is an exemplification of the size distribution of a given formulation that helps in deciding whether the suspended elements are homogeneous (≤0.3) or heterogeneous (>0.3) in nature [51].

The PVA plays a substantial role in deciding the particle size range because an increased amount of PVA improves the viscosity of the medium, thus reducing the shear stress, which is an essential variable in the reduction of particle size [36,48]. Furthermore, PVA adheres to the surface of nanosponges and continues to adhere to them even after repeated washings, resulting in the growth of particle size [46,56].

The generated nanosponges had a PDI value within an acceptable range (0.3890 ± 0.0943); however, if the value exceeds 0.7, the DLS analysis could not be completed due to the high degree of variability in the size distribution [52]. 

#### 2.1.5. Entrapment Efficiency (%EE)

The EE is usually associated with the modification in the various formulation aspects that affect the ability of the nanosponges to hold a drug molecule [56]. MGN loaded nanosponges exhibited an amicable production yield (75 ± 11%) and entrapment efficiency (89 ± 5%) as shown in Table 1. Higher EE is associated with a slow release of the entrapped drug and quite similar results were exhibited by MGN nanosponges where the ratio of EC and PVA was optimized as 2:1. An optimal quantity of PVA is highly desirable in the nanosponge formation [53].

#### 2.1.6. In Vitro Dissolution Release and Release Kinetics

The release behavior of MGN loaded nanosponges displayed a controlled release of MGN (94% in 12 h) as shown in Figure 2D. Various pharmacokinetic models including zero-order, Higuchi model, first-order, and Korsmeyer Peppas were applied on release profile data using DDSolver to elucidate the MGN release pattern from prepared nanosponges. The values of the regression coefficients for each model are listed in Table 1.

The hydrophobic nature of EC as well as phase transition largely influenced the release kinetics of MGN over an extended time duration (12 h). The cumulative release of nanosponges having MGN (1:1) in 12 h was 94% suggesting that MGN was released in a controlled manner. The results were best suited by the Higuchi model, which had a regression coefficient (R^2^) of 0.9121, indicating that drug molecules were distributed equally inside the matrix of nanosponges. The Higuchi model was observed as the best fit depicting the value of 0.9121 for the regression coefficient indicating uniform dispersal of drug metabolism throughout nanosponges. Concentration-dependent release kinetics was shown by regression data from zero-order release (0.793) as well as Korsmeyer Peppas (0.9304; n = 0.497) and first-order (0.9959). The first-order release behavior was supported by aforesaid results whereas the “n” value showed release following non-fickian in which diffusion as well as erosion and swelling both are responsible for drug release [33,44,55,56].

### 2.2. In Vivo Studies

In vivo studies were conducted on male Wistar rats by strictly adhering to the guidelines as approved by Pharmacy Ethical Committee (12/PEC/2019), Faculty of Pharmacy, Bahauddin Zakariya University, Multan, Pakistan. Diabetes was induced in the rats by intraperitoneal injection of streptozotocin (60 mg/kg body weight) [57]. Plasma glucose, as well as MGN levels, were determined in different animal groups following oral administration of MGN (as free dispersion) and MGN loaded nanosponges using the same dose. A rapid hypoglycemic response was observed upon administration of pure MGN with a maximum response of 28.71% (67.13 ± 4.924 mg/dL blood glucose level *p* = 0.0032) at T_max_ of 1 h. 

**Figure 3:**
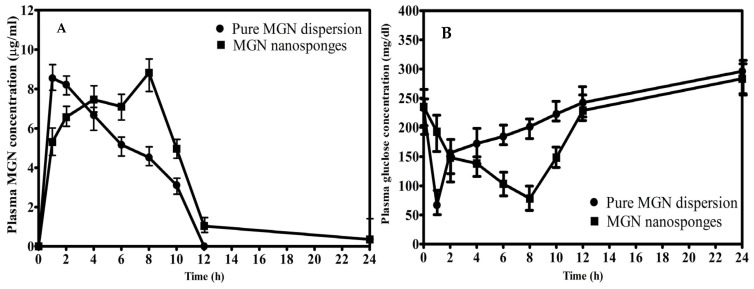
Plasma concentration in experimental rats after administration of pure MGN and MGN nanosponges (**A**), and plasma glucose concentration in experimental rats after administration of pure MGN and MGN nanosponges in different time intervals (**B**).

A comparatively steady hypoglycemic response was observed with MGN loaded nanosponges with a T_max_ of 8 h and a maximum response of 33.35% with a blood glucose level of 78.42 ± 11.52 mg/dL (*p* = 0.0028) following oral administration. A significant increase in AUC_0-12_ besides T_max_ and the hypoglycemic response was observed upon oral administration of MGN loaded nanosponges as evident from statistical data (independent samples *t*-test) in comparison to pure MGN (*p* < 0.05). The larger hypoglycemic response observed for MGN loaded nanosponges was due to a higher penetrating ability of drug encapsulated via hydrophobic moiety. Our findings (Figure 3 and Table 2) were in accordance with the previous reports [12,16,54,58]. When free nanosponges were given to diabetic rats, the animals expired as a result of acute hyperglycemia, demonstrating that the excipients (EC and PVA) were inert and had no role in lowering plasma glucose levels. Our results were consistent with the outcomes found in enzyme assay where free nanosponges showed no inhibitory potential against α-glucosidase.

### 2.3. In Vitro Enzyme Inhibition Studies

#### α-Glucosidase Inhibitory Activity

**Figure 4:**
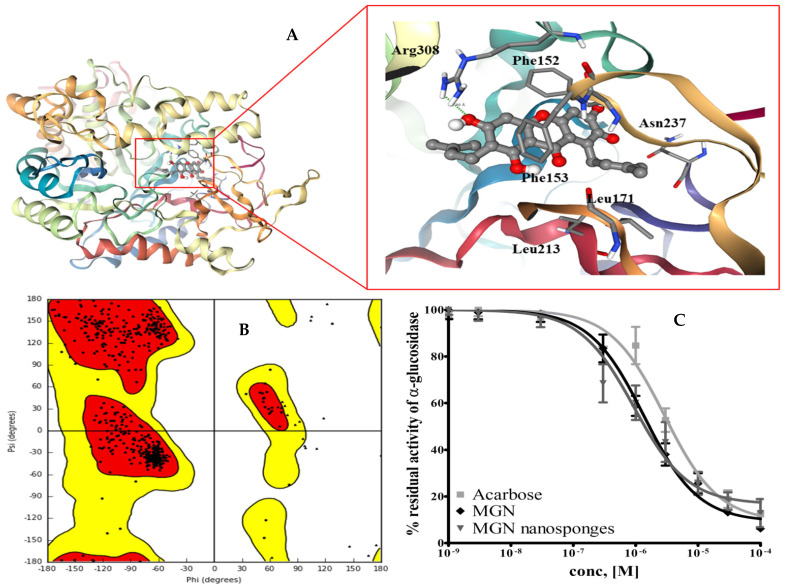
The simulated binding mode of the MGN in the binding site of yeast α-glucosidase. The ligand is presented in a ball and stick model with the hydrogen bond represented with dashed lines. The graphic was rendered using NGL viewer (**A**). The Ramachandran plot of the developed model. The core and outer contours present the allowed and the generously allowed regions (**B**). Inhibition studies of MGN nanosponges against α-glucosidase with IC_50_ values of 1.353 µM (MGN), 0.9352 µM (MGN nanosponges), and 2.909 µM (acarbose) (**C**).

Enzyme inhibition studies were carried out initially at a single concentration of 10 mM and further, a three-fold serial dilution of each inhibitor was made to estimate IC_50_ (Figure 4C). Both pure MGN and its nanosponges, as well as free nanosponges, were tested against α-glucosidase [53]. The obtained data were compared with the standard inhibitor, acarbose. The maximum inhibition against α-glucosidase was observed by nanosponges (0.9352 ± 0.0856 µM) that was 1.44-fold more potent than pure MGN (1.353 ± 0.3751 µM) and 3.11-fold more potent than acarbose, the standard inhibitor (ANOVA test where *p* < 0.05). Our results corroborated earlier reports of mangostin acting as an α-glucosidase inhibitor [13,59,60]. When tested against α-glucosidase inhibition, the free nanosponges were ineffective, which confirmed their inert nature.

### 2.4. Molecular Docking Studies

To establish the protein-ligand contact profile of MGN and α-glucosidase complex, molecular docking simulations were carried out. For this purpose, the homology model of the *S. cerevisiae* α-glucosidase was developed using the SWISS-MODEL web-server [61]. The isomaltase from the same species (PDB: 3AJ7) was used as a template. The stereochemical quality of the model was accessed with the help of the Ramachandran plot (Figure 4A) [62]. As evident from the graph, more than 97% of the residues lie in the allowed region which underpins the reliability of the developed model.

To establish the binding mode, the MGN was subjected to molecular docking studies using the homology modeled structure of the yeast α-glucosidase. The pose obtained from docking was refined with the help of MD simulation. Figure 4B presents the post-MD simulation pose of the MGN in the binding site of the *S. cerevisiae* α-glucosidase. As evident from Figure 4, MGN resides comfortably in the binding site of α-glucosidase facilitating contacts with the neighboring residues. The xanthone core structure mediates π-stacking interaction with Phe152 and Phe153 at the binding site. The Phe152 and Phe153 are part of the hydrophobic patch of α-glucosidase that facilitates the catalysis by stacking the face of the saccharide rings. Apart from the stacking interactions, MGN exhibits hydrophobic contacts with the aliphatic chains of the Leu171, Leu213, and Asn237. It has been reported that these hydrophobic interactions are the fundamental interactions responsible for protein-ligand complexation [63]. In addition, our findings revealed that MGN forms a hydrogen connection with the guanidinium cap of the Arg308 molecule. 

## 3. Materials and Methods

### 3.1. General

The α-mangostin (MGN), ethyl cellulose (EC), dichloromethane (DCM), polyvinyl alcohol (PVA), α-glucosidase, acarbose, streptozotocin, phosphate-buffered saline (PBS) tablets, potassium bromide (KBr), lysozyme, dialysis membrane (10K MWCO), diethyl ether, and ethanol were procured from Merck KGaA (St. Louis, MO, USA) and were utilized without any additional purification.

### 3.2. Animals

Adult male Sprague Dawley rats (*Rattus norvegicus*) with an average weight of 270–290 g were obtained from the animal house facility at Faculty of Pharmacy, Bahauddin Zakariya University, Multan, Pakistan, and were harbored in appropriate cages and familiarized with the laboratory environment for at least one week before the start of experiments. The test animals were nurtured day and night with standard rodent food and mineralized water. The study design was approved by the committee on animal and research ethics of Bahauddin Zakariya University, Multan, Pakistan.

### 3.3. Development of MGN Nanosponges 

Solvent evaporation technique was utilized to formulate MGN entrapped nanosponges with slight modifications [55]. Briefly, MGN and ethyl cellulose were dissolved in 2:1 molar ratios in dichloromethane (20 mL) while the aqueous phase was comprised of PVA (0.4%). The organic phase was added slowly into the aqueous phase. Subsequently, the mixture was stirred properly at 20000 rpm for 2 h to form nanosponges. The MGN nanosponges were dried into a powder and kept in the refrigerator until further use.

### 3.4. Physical Characterization of Nanosponges

#### 3.4.1. Fourier Transform Infra-Red (FTIR) Spectroscopic Analysis

KBr disc method was used to obtain the spectra of pure MGN, MGN nanosponges, and free nanosponges recorded on a Shimadzu IR-Prestige FTIR spectrophotometer (Shimadzu IRPrestige-21 Tokyo, Japan) across a range of 4000 to 500 cm^−1^ [64].

#### 3.4.2. Differential Scanning Calorimetric (DSC) Analysis

The physical stability was evaluated with thermal analysis of pure MGN, MGN nanosponges, and blank nanosponges. The heating rate was raised at 10 °C/min together with an uninterrupted nitrogen flow (2 mL/min) to prevent oxidation. The thermogram was produced using DSC 214 Polyma (NETZSCH Instruments, Burlington, USA) by heating to 200 °C using a hollow aluminum pan as a reference [44].

#### 3.4.3. Scanning Electron Microscopic (SEM) Analysis

SEM images of MGN nanosponges were obtained using a Hitachi S-4700 (Houghton, MI, USA) with an acceleration voltage of 10–20 kV. The sample was dispersed in ethanol and instantaneously placed on perfect silicon wafers. For smooth conduction, the sample was sputter-coated with gold [65].

#### 3.4.4. Particle Size Estimation

To determine the hydrodynamic size distribution of MGN nanosponges, the dynamic light scattering (DLS) approach was used. The MGN nanosponges were dispersed in double-distilled water for DLS analysis. A Malvern Zetasizer Nano ZS (Cambridge, UK) equipment was used to determine the zeta potential [66].

#### 3.4.5. Determination of Entrapment Efficiency (%EE)

The entrapment efficiency (%EE) of MGN in nanosponges was calculated as reported previously [67]. Briefly, a dialysis bag containing 5 mL of nano-dispersion was submerged in PBS (100 mL). The setup was placed on a magnetic stirrer (75 rpm) at 37 °C for 1 h. The sample was appropriately diluted before measuring absorbance at 262 nm using a UV-visible spectrophotometer (Shimadzu, Tokyo, Japan). The percent EE was calculated using the following equation [56]:

(1) $$\mathrm{Entrapment}\;\mathrm{Efficiency}\;\left(\%\mathrm{EE}\right)=\left[\frac{Total\;amount\;of\;MGN\;in\;nanosponges-Free\;MGN}{Total\;amount\;of\;MGN\;in\;nanosponges}\right]\times 100$$

#### 3.4.6. Determination of Production Yield (%)

The MGN nanosponges obtained after drying were weighed. Percentage yield value was calculated as follows [68]:

(2) $$\mathrm{Production}\;\mathrm{yield}\;\left(\%\right)=\left[\frac{\mathrm{Weight}\;\mathrm{of}\;\mathrm{nanosponges}}{\mathrm{Total}\;\mathrm{amount}\;\mathrm{of}\;\mathrm{solid}\;\mathrm{ingredients}}\right]\times 100$$

#### 3.4.7. In Vitro Dissolution Studies

The MGN release pattern and kinetic models were studied based on the previously reported method [67]. Briefly, the dialysis membrane (10K MWCO) was filled with nanosponge dispersion (10 mg in 5 mL of PBS) and tied on both ends. The dialysis tube was immersed in 0.1M HCl (250 mL) for 2 h initially and then transferred to PBS (250 mL, pH 6.8) with lysozyme (0.6 g/mL). The experiment was conducted on a magnetic stirrer set at 37 °C with 75 rpm. The samples were collected at predetermined intervals, while MGN release was estimated on a UV-Visible spectrophotometer (Shimadzu, Tokyo, Japan). The acquired data were examined using the DDsolver software for drug release behavior utilizing zero-order, first-order, Higuchi, and Korsmeyer–Peppas models.

### 3.5. In Vitro Enzyme Inhibition Studies

#### α-Glucosidase Inhibitory Activity

The α-glucosidase assay was performed using a previously reported method with minor alterations [69]. Briefly, the reaction mixture comprised of 50 µL of enzyme solution (0.4 U/mL) and 50 µL of *p*-nitrophenyl-α-d-glucopyranoside (pNPG, 1 mM) as substrate was prepared in sodium phosphate buffer (pH 6.8) with the addition of a 10 µL of the test sample. The reaction was terminated by adding 0.1 M NaOH. The control (acarbose) and blank (negative control) wells were also maintained in a 96 well-plate for analysis. The α-glucosidase activity was determined by measuring the extent of hydrolysis of pNPG and estimating the formation of *p*-nitrophenol measured at 405 nm using ELISA microplate reader ELx808™ (BioTek Instruments, Winooski, VT, USA). The experiment was performed in triplicate and data was presented with the standard error of the mean (SEM). The formulations showed ≥50% enzyme inhibition were further used to calculate IC_50_ value by using GraphPad Prism5 software.

### 3.6. In Vivo Anti-Diabetic Activity 

#### 3.6.1. Induction of Type 2 Diabetes in Rats

T2DM was instigated in overnight starving rats with an intraperitoneal (i.p) injection of streptozotocin (65 mg/kg) dissolved in citrate buffer (pH 4.5). After 72 h of diabetes induction, the rats with persistent high glucose levels (>200 mg/dL) were considered diabetic and included in the study [70].

#### 3.6.2. Experimental Design and Blood Sampling

Healthy male rats were randomly divided into five groups where each group contains five animals and received treatment orally. Among ten, Group I was considered as the control which received the standard anti-diabetic treatment with acarbose while Group II was based on healthy rats that received distilled water orally. Group III was given pure MGN (equivalent to pre-determined IC_50_) as a test compound while MGN nanosponges (equivalent to IC_50_) were administered to Group IV. Group V was evaluated to see if the excipients produced the desired hypoglycemic response in diabetic rats by giving free nanosponges.

At specified time intervals (1, 2, 3, 4, 6, 8, 10 and 12 h), the animals were sacrificed after giving anesthesia with diethyl ether and blood was collected into dry clean EDTA containing test tubes. Blood plasma samples were run on HPLC to determine the concentration of free MGN and MGN nanosponges through pharmacokinetic analysis [71,72].

#### 3.6.3. HPLC Assay Method

A 600 µL of blood was removed from rats under investigation and centrifuged at 10,000 rpm for 5 min. The isolated plasma (300 µL) was incubated with methanol (300 µL) to induce protein precipitation. Afterward, the mixture was shaken gently and again centrifuged at 10,000 rpm for 5 min. The supernatant was filtered and diluted with 100 µL of the mobile phase, from which a 20 µL was taken into HPLC to determine the concentration of MGN. 

The conditions for the HPLC assay were as follows: The HPLC-LC20A system (Shimadzu, Tokyo, Japan) consisted of an LC-10AT pump, SPD-A20 UV-Vis detector, SIL-20A/C autosampler, and Shimadzu LC-solution software. Chromatographic separation of MGN was achieved by using a Shim-pack MAqC-ODS (150 mm × 4.6 mm × 5 μm) reverse-phase analytical column at ambient temperature. The mobile phase consisted of ammonium acetate (20 mM, pH 6.8) and methanol (5%). An isocratic elution strategy was adopted with a flow rate of 0.5 mL/min. The concentration of eluate (MGN) was calculated and plotted against time using Prism5 software. The pharmacokinetic parameters, area under the concentration-time curve (AUC), maximal response, and period of maximal response were investigated (T_max_). The in vivo results were reported as SEM (standard error of the mean) [58].

### 3.7. Molecular Docking Studies

To establish the plausible protein-ligand interaction profile of the MGN and α-glucosidase complex, molecular docking simulations were carried out using a homology model of *S. cerevisiae* α-glucosidase. The SWISS-MODEL web-server was used to develop a homology model using the isomaltase from the same species as a template [73]. The stereochemical quality of the model was assessed by plotting the Ramachandran plot of the Phi and Psi angles. The system was then prepared for docking calculations using the AMBER10: EHT force field implied in the MOE software suite (Chemical computing group, Cambridge, UK).

To benchmark the ability of software to reproduce the crystal pose; the re-docking experiment was carried out using the Protein Data Bank (PDB); 3A4A. After successful re-docking results, MOE was used to establish the binding mode of the MGN. The binding site was identified using the coordinates of the yeast isomaltase complex. The default placement and scoring methods were used in combination with the rigid receptor docking protocol in MOE. The poses generated by the initial placement and scoring methods were subjected to refinement and rescoring. The top-ranked pose was then subjected to post-docking minimization using the MD simulation protocol developed earlier [26]. A short production run of 10 ns was carried out, and the post-MD pose was visually analyzed. 

### 3.8. Statistical Analysis

The results were evaluated using independent samples *t*-test (pharmacokinetics analysis) and one-way analysis of variance (enzyme inhibition assay), with a 95% confidence interval (*p* < 0.05). Certain experimental results, however, were represented as the standard error of the mean (SEM). Microsoft Excel (2010),.(Microsoft Corp, Redmond, WA, USA), SPSS (9.0), (SPSS Inc. Chicago, IL, USA) and Prism (5.0) (GraphPad Software, San Diego, CA, USA) software were used for the statistical analysis.

## 4. Conclusions

In summary, the present study describes the fabrication of MGN nanosponges for anti-diabetic therapeutic applications. MGN nanosponges were characterized by Fourier-transform infra-red (FTIR) spectroscopy, differential scanning calorimetry (DSC), scanning electron microscopy (SEM), zeta potential, hydrodynamic diameter, entrapment efficiency, controlled drug release, and stability studies at stress conditions. The results demonstrate that the use of MGN loaded nanosponges as a drug delivery system for oral administration would be beneficial since when compared to free drug solution given at the same dosage, a longer and enhanced hypoglycemic impact may be achieved. Thus, the number of dosages that must be administered to patients each day is reduced, and it is anticipated that the number of adverse effects associated with the medication would be reduced as well. This allows for the creation of a convenient dose form. Taken together, the MGN loaded nanosponges described here may function as an archetype for natural materials traditionally used for diabetes treatment. This study may uncover further prospects and exhaust the potential of nutraceuticals.

## Figures and Tables

**Table 1:** Physical characterization and kinetic models of MGN nanosponges.

Properties/Models	Outcomes
Zeta Potential	−35.06 ± 4.91 mV
PDI	0.3890 ± 0.0943
Entrapment Efficiency	89 ± 5 (%)
Production Yield	75 ± 11 (%)
Hydrodynamic Diameter	113 ± 8 nm
Zero-Order	0.7935
First-Order	0.9959
Higuchi Model	0.9121
Korse–Meyer Peppas, n Value	0.9304, 0.4970

**Table 2:** Data of in vivo pharmacokinetic model of pure and MGN loaded nanosponges.

		Parameters of Activity					
Formulation		$AUC_{0-12}$ (mg.h/dL) ± SEM			Max. Hypoglycemic Response (mg/dL) ± SEM		$T_{max}\;(h)$
Pure MGN Dispersion		233.8 ± 15.31			67.13 ± 4.925		1
MGN Nanosponges		235.1 ± 17.62			78.42 ± 11.52		8
		**Glucose Concentration** **(mg/dL) ± SEM**			**Plasma MGN Concentration** **(µg/mL) ± SEM**		
**Sr.No**	**Groups Description**	**Pure MGN Dispersion**	**MGN Nanosponges**	***p*-value**	**Pure MGN Dispersion**	**MGN Nanosponges**	***p*-value**
1	Normal Control	85.64 ± 9.356	87.11 ± 6.579	0.8149	---	---	---
2	Diabetic Control	233.8 ± 15.31	235.1 ± 17.62	0.9736	---	---	---
3	After 1 h	67.13 ± 4.925	192.8 ± 20.71	0.0032	8.551 ± 2.689	5.307 ± 2.851	0.0384
4	After 2 h	156.8 ± 18.61	148.7 ± 24.91	0.4271	8.201 ± 1.662	6.568 ± 1.897	0.1254
5	After 4 h	172.4 ± 15.84	136.6 ± 15.74	0.1845	6.679 ± 3.415	7.462 ± 3.644	0.4918
6	After 6 h	184.7 ± 19.84	103.1 ± 15.32	0.0391	5.162 ± 1.204	7.108 ± 1.927	0.7612
7	After 8 h	201.5 ± 18.69	78.42 ± 11.52	0.0028	4.508 ± 1.691	8.824 ± 2.607	0.0064
8	After 10 h	223.1 ± 17.96	148.5 ± 16.71	0.0414	3.117 ± 1.141	4.971 ± 1.845	0.0217
9	After 11 h	242.6 ± 26.53	229.1 ± 18.24	0.4628	Not detected	1.035 ± 0.360	0.0138
10	After 12 h	296.2 ± 27.38	283.7 ± 31.10	0.4773	Not detected	0.352 ± 0.028	0.0413

The *p*-value of <0.05 will be considered statistically significant.

## Data Availability

The data presented in this study are available from the authors on reasonable request.

## Sample Availability

Samples of the compounds (α-mangostin nanosponges) are available from the authors.
